# Identification and selective expansion of functionally superior T cells expressing chimeric antigen receptors

**DOI:** 10.1186/s12967-015-0519-8

**Published:** 2015-05-20

**Authors:** ZeNan L. Chang, Pamela A. Silver, Yvonne Y. Chen

**Affiliations:** Department of Chemical and Biomolecular Engineering, University of California–Los Angeles, 420 Westwood Plaza, Boelter Hall 5531, Los Angeles, CA 90095 USA; Molecular Biology Institute, University of California–Los Angeles, 611 Charles E. Young Drive East, Boyer Hall 168B, Los Angeles, CA 90095 USA; Department of Systems Biology, Harvard Medical School, 200 Longwood Avenue, WAB 563, Boston, MA 02115 USA

**Keywords:** Chimeric antigen receptor, CD19 CAR-T cell, T-cell immunotherapy, PD-1

## Abstract

**Background:**

T cells expressing chimeric antigen receptors (CARs) have shown exciting promise in cancer therapy, particularly in the treatment of B-cell malignancies. However, optimization of CAR-T cell production remains a trial-and-error exercise due to a lack of phenotypic benchmarks that are clearly predictive of anti-tumor functionality. A close examination of the dynamic changes experienced by CAR-T cells upon stimulation can improve understanding of CAR–T-cell biology and identify potential points for optimization in the production of highly functional T cells.

**Methods:**

Primary human T cells expressing a second-generation, anti-CD19 CAR were systematically examined for changes in phenotypic and functional responses to antigen exposure over time. Multi-color flow cytometry was performed to quantify dynamic changes in CAR-T cell viability, proliferation, as well as expression of various activation and exhaustion markers in response to varied antigen stimulation conditions.

**Results:**

Stimulated CAR-T cells consistently bifurcate into two distinct subpopulations, only one of which (CAR^hi^/CD25^+^) exhibit anti-tumor functions. The use of central memory T cells as the starting population and the resilience—but not antigen density—of antigen-presenting cells used to expand CAR-T cells were identified as critical parameters that augment the production of functionally superior T cells. We further demonstrate that the CAR^hi^/CD25^+^ subpopulation upregulates PD-1 but is resistant to PD-L1-induced dysfunction.

**Conclusions:**

CAR-T cells expanded *ex vivo* for adoptive T-cell therapy undergo dynamic phenotypic changes during the expansion process and result in two distinct populations with dramatically different functional capacities. Significant and sustained CD25 and CAR expression upregulation is predictive of robust anti-tumor functionality in antigen-stimulated T cells, despite their correlation with persistent PD-1 upregulation. The functionally superior subpopulation can be selectively augmented by careful calibration of antigen stimulation and the enrichment of central memory T-cell type.

**Electronic supplementary material:**

The online version of this article (doi:10.1186/s12967-015-0519-8) contains supplementary material, which is available to authorized users.

## Background

Adoptive cell therapy using T cells engineered to express tumor-targeting chimeric antigen receptors (CARs) is a promising treatment strategy for refractory diseases such as metastatic melanoma, leukemia, and neuroblastoma [1–3]. Several recent trials have demonstrated remarkable clinical efficacy, particularly in the treatment of chronic and acute B-cell malignancies using CD19-targeting T cells [4–6]. Accumulating clinical reports suggest that, in addition to patient-to-patient variations in tumor burden and overall health profile, the quality of individual T-cell products could significantly influence clinical outcome [5, 7]. However, it remains unclear which T-cell characteristics are the most critical and predictive of anti-tumor efficacy, and if and how such characteristics could be promoted during T-cell manufacturing. As CAR-T cell technology advances toward broader clinical use, the ability to identify critical T-cell characteristics and systematically optimize CAR-T cell preparation has the potential to significantly improve the robustness of adoptive T-cell therapy.

Considerable differences exist in the T-cell manufacturing protocols utilized thus far by different clinical groups, rendering efforts to systematically optimize the production process a practical challenge. Although numerous characterization assays and product release criteria are applied to CAR-T cell products, they typically generate snapshot views of T-cell properties and provide limited insight into dynamic changes experienced by T cells, which may occur throughout *in vitro* expansion as well as after infusion into the patient. For example, phenotypic characteristics such as % CD3^+^, % CD4^+^, % CD8^+^, and % CAR^+^ are typically quantified at the end of cell expansion prior to product release for infusion [4–6, 8]. Cytokine production and cell lysis efficiency are measured *in vitro* at single time points to confirm target-specific functional activity [5, 6, 9]. After adoptive transfer, *in vivo* performance is measured by quantifying cytokine levels, tumor burden, and CAR^+^ T-cell count in the patient [4, 10, 11]. In these characterization assays, observed anti-tumor functionality is attributed to CAR^+^ T cells as a homogenous group, and time-point data are used to generalize across cell-expansion and treatment periods. Given that current clinical protocols typically utilize unsorted, polyclonal T cells for infusion, the assumption of uniformity among CAR^+^ T cells is one dictated by experimental constraints rather than our understanding of CAR–T-cell biology. Indeed, the recognition that not all T cells are equal has prompted active research on questions such as the optimal T-cell subtype and cytokine regimen to use for the production of therapeutic T cells [12–16]. However, trial-and-error remains the dominant approach to process optimization, as typical characterization methods such as those described above provide information that enables quality control but not in-depth understanding of how the T cells arrived at their present state of functionality or lack thereof. We propose that a close examination of dynamic changes experienced by CAR-T cells throughout a stimulation cycle can provide a deeper understanding of CAR–T-cell biology and identify potential points for optimization in the production of highly functional therapeutic T cells.

In this study, we perform quantitative evaluations of the phenotypic and functional changes exhibited by CAR-T cells undergoing antigen stimulation, including CAR–T-cell viability, proliferation, as well as the expression of various T-cell activation and exhaustion markers. Contrary to the assumption of uniformity, stimulated CAR^+^ T cells consistently bifurcate into two distinct populations, only one of which (CAR^hi^/CD25^+^) is functionally active. Detailed *in vitro* examinations reveal dynamic changes in CAR-T cells over the course of antigen stimulation that are difficult to observe *in vivo*, and enable the identification of strategies to maximize the CAR^hi^/CD25^+^ cell subpopulation upon antigen stimulation. Finally, we demonstrate that CAR-T cells in the functionally superior subpopulation upregulate PD-1 but remain functional when challenged by target cells overexpressing PD-L1, indicating an unexpected source of CAR-T cell resilience and highlighting properties to be considered in the development of combinatorial strategies employing CAR-T cells and checkpoint therapies.

## Methods

### Cell line isolation and maintenance

Primary human CD4^+^ or CD8^+^ T cells were isolated from healthy donor blood samples obtained from the Boston Children’s Hospital Blood Donor Center and the UCLA Blood & Platelet Center. The RosetteSep CD4^+^ or CD8^+^ Human T-cell Enrichment Cocktail (Stemcell Technologies) was used following manufacturer’s protocols. Central memory T cells were obtained by magnetic bead-based sorting. Anti-CD45RA MicroBeads (Miltenyi Biotec) were used to deplete CD45RA^+^ cells, and CD45RA^−^ cells were stained with anti-CCR7-APC (clone G043H7, BioLegend) followed by anti-APC MicroBeads (Miltenyi Biotec) and enriched for CCR7^+^ cells. Isolated T cells were seeded at 1 × 10^6^ cells/ml in T-cell media (RPMI-1640 media (Lonza) with 10 % heat-inactivated fetal bovine serum (FBS; Life Technologies)) and stimulated with CD3/CD28 Dynabeads (Life Technologies) at a 1:1 cell:bead ratio. Cells were fed 50 IU/ml interleukin (IL)-2 (Chiron) and 1 ng/ml IL-15 (Miltenyi Biotec) every 48 h and passaged routinely. H9 and JeKo-1 cells were obtained from ATCC and maintained in RPMI-1640 with 10 % or 20 % heat-inactivated FBS, respectively. K562, TM-LCL, and Raji cells were generous gifts from Dr. Michael C. Jensen at Seattle Children’s Research Institute and maintained in T-cell media. CD19^+^ K562 and CD19^+^ H9 cells were generated by lentivirally transducing parental K562 and H9 cells with CD19 cDNA expressed from an EF1α promoter and sorting for CD19^+^ cells by fluorescence-activated cell sorting (FACS).

### Generation and isolation of CAR-expressing T cells

Lentivirus was produced as previously described [17]. T-cells stimulated with CD3/CD28 Dynabeads for 3 days were transduced at a multiplicity of infection of 3 with 1 × 10^6^ T cells/500 μl/well in a 24-well plate and supplemented with 50 IU/ml IL-2, 1 ng/ml IL-15, and 5 μg/ml polybrene (Sigma Aldrich). No virus was added to the mock-transduced control. The plate was centrifuged at 800 × *g* for 30 min at room temperature with slow acceleration and no brake. Cells were fed fresh media with cytokines on day 2 post transduction, washed on day 3, and maintained as described above until Dynabead removal on day 6 post transduction. To obtain EGFRt^+^ (CAR^+^) populations, transduced cells were stained with biotinylated Erbitux (Bristol-Myers Squibb; biotinylated in house) followed by magnetic sorting using anti-Biotin MicroBeads (Miltenyi Biotec) according to the manufacturer’s protocols. CAR^+^ T-cell fractions with different CAR expression levels were isolated by staining transduced cells with biotinylated Erbitux followed by streptavidin-PE (Jackson Immunoresearch), then sorted by FACS. Regardless of sorting method, CAR^+^ cells were expanded as previously described [18]. Briefly, 1 × 10^6^ T cells were resuspended in 50 ml total volume with 7 × 10^6^ γ-irradiated (80 Gy) TM-LCL cells and supplemented with 50 IU/ml IL-2 and 1 ng/ml IL-15 every 48 h. Stimulated high and low CAR-expressing populations were isolated by FACS after 20 h of co-incubation with CD19^+^ K562 target cells at a 2:1 effector-to-target (E:T) ratio.

### Surface marker staining

For surface marker staining, 1 × 10^5^ T cells were seeded in 96-well plates with indicated target cells (unirradiated) at a 2:1 E:T ratio unless otherwise noted. Experiments with 2.5 × 10^5^ T cells were performed in 24-well plates. When indicated, γ-irradiated (100 Gy) K562 targets were used. When indicated, CD28 monoclonal antibody (clone CD28.2; eBiosciences) was applied at 10 μg/mL to provide CD28 costimulatory signal. Cell mixtures were incubated at 37 °C, and analyzed at the indicated time points with fluorescently labeled monoclonal antibodies binding CCR7 (clone REA108), CD19 (clone LT19), CD25 (clone BC96), CD27 (clone M-T271), CD45RA (clone T6D11), CD57 (clone TB03), PD-1 (PD1.3.1.3), PD-L1 (clone 29E.2A3), and Tim-3 (clone F38-2E2) (BioLegend and Miltenyi Biotec). V500-conjugated Annexin V (BD Biosciences) and Pacific Blue-conjugated Annexin V (BioLegend) were used to detect pre-apoptotic cells. CAR expression was probed with Protein L (Genscript) followed by PE-conjugated streptavidin (Jackson Immunoresearch) or with APC-conjugated polyclonal antibody binding human IgG Fcγ (Jackson Immunoresearch). EGFRt expression was probed with biotinylated Erbitux followed by PE-conjugated streptavidin. Analyses were performed on a MACSQuant VYB flow cytometer (Miltenyi Biotec) equipped with 405-, 488-, and 561-nm lasers. Data were processed using FlowJo software (TreeStar).

### Cell proliferation and cytokine production quantification

T cells were labeled with 0.2 μM carboxyfluorescein diacetate succinimidyl ester (CFDA-SE, Life Technologies) for cell proliferation tracking. A human Th1/Th2 cytokine cytometric bead array kit (BD Biosciences) was used according to the manufacturer’s protocols to quantify cytokine secretion. Co-incubations with target cells were set up as described above. Samples were analyzed with a MACSQuant VYB flow cytometer and cytokine production was quantified using the FCAP Array 3.0 software (Soft Flow).

### Quantitative PCR

Genomic DNA and cDNA were isolated from frozen T-cell pellets with DNeasy Blood and Tissue Kit (Qiagen) and SuperScript III CellsDirect cDNA Synthesis System (Life Technologies), respectively. Quantitative PCR (qPCR) was performed using SsoFast EvaGreen Supermix (Bio Rad), a CFX96 Real-Time Thermal Cycler (Bio Rad), and WPRE forward and reverse primers (5′-TTTCCGGGACTTTCGCTTTC and 5′-AAGGGACGTAGCAGAAGGAC, respectively, for cDNA; or 5′-ACTGTGTTTGCTGACGCAACCC and 5′-CAACACCACGGAATTGTCAGTGCC, respectively, for gDNA) according to the manufacturer’s protocols. The reference β-actin primer set 5′-TCCCTGGAGAAGAGCTACGA (forward) and 5′-AGCACTGTGTTGGCGTACAG (reverse) provided normalization control. The qPCR protocol included 30 cycles of a 5-s denaturation step at 95 °C for cDNA and 98 °C for gDNA, and a 5-s annealing/extension step at 60 °C. All reactions were performed in quadruplicates, and threshold cycle (Ct) values were averaged to obtain the arithmetic mean. Relative WPRE levels were calculated with the following formula:

$$ RL=\frac{{\left({\varepsilon}_{Actin}\right)}^{C_{t,\  Actin}}}{{\left({\varepsilon}_{WPRE}\right)}^{C_{t,\  WPRE}}} $$

where RL indicates relative WPRE levels, ε_x_ indicates primer efficiency for gene x, and C_t,x_ indicates the averaged Ct value for gene x. Standard deviation in relative WPRE levels was calculated with the following formula:

$$ s.d.=\sqrt{{\left(RL\kern0.5em  \ln {\varepsilon}_{Actin}\right)}^2{\left(s.d{.}_{Actin}\right)}^2+{\left(RL\kern0.5em  \ln {\varepsilon}_{WPRE}\right)}^2{\left(s.d{.}_{WPRE}\right)}^2} $$

where s.d._x_ indicates the standard deviation calculated from the quadruplicate samples for gene x.

### Statistical methods

Data are presented as means ± standard deviations as stated in figure legends. Results were analyzed by two-tailed unpaired Student’s *t* test with simple Bonferroni correction for multiple comparisons when appropriate. Tests were conducted with statistical significance set at *p* < 0.05.

## Results

### Antigen stimulation results in the emergence of CAR^hi^ cells

**Fig. 1:**
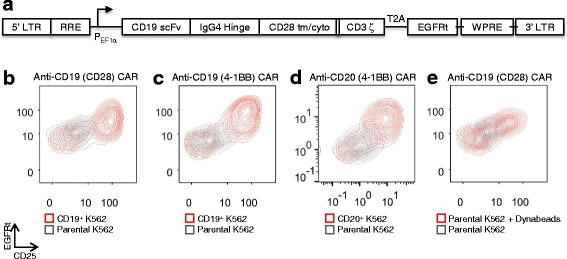
A CAR^hi^/CD25^+^ population emerges upon antigen stimulation of CAR-T cells. **a** Schematic of the second-generation anti-CD19 CAR with CD28 co-stimulatory domain; cyto: cytoplasmic domain; EGFRt: truncated EGFR peptide; LTR: long terminal repeats; RRE: Rev responsive element; tm: transmembrane domain; scFv: single chain variable fragment; T2A: *Thosea asigna* virus 2A peptide; WPRE: woodchuck hepatitis virus posttranscriptional response element. **b-e** CD8^+^ T cells expressing various CARs separate into CAR^hi^/CD25^+^ and CAR^lo^/CD25^−^ populations within 24 h of antigen or CD3/CD28 stimulation. T cells expressing anti-CD19 CARs with **b** CD28 or **c** 4-1BB co-stimulation domain or **d** an anti-CD20 CAR with 4-1BB all result in population polarization upon stimulation with K562 target cells expressing cognate antigens. **e** CAR–T-cell stimulation with magnetic beads coated with anti-CD3 and anti-CD28 antibodies also yields bifurcated populations

To investigate the degree of heterogeneity among CAR-T cells, CAR constructs were stably integrated into bulk primary human CD8^+^ T cells via lentiviral transduction. Unless otherwise indicated, an anti-CD19 CAR containing CD28 as the co-stimulatory domain was used [19], and a truncated epidermal growth factor receptor (EGFRt) was linked to the CAR via a T2A peptide (Fig. 1a). In this configuration, CAR and EGFRt are transcribed as a single mRNA but translated into two separate proteins, allowing quantification of CAR expression via antibody staining of surface-bound EGFRt, without disrupting potential interactions between CAR molecules and their ligands [20]. Co-staining experiments confirmed that EGFRt staining tightly correlates with direct CAR staining in both stimulated and unstimulated CAR-T cells (Additional file 1: Figure S1). EGFRt is particularly useful to track the expression of the anti-CD19 CAR in this study because the anti-CD19 scFv used is only weakly stained by reagents such as protein L and anti-Fab antibody that typically bind scFvs. Unless specified otherwise, we have used EGFRt to measure CAR expression in the studies reported here.

CAR-T cells were co-incubated with parental (CD19^−^) or CD19^+^ K562 target cells and monitored for five days. A CAR^hi^/CD25^+^ group with elevated CAR expression consistently emerged within 24 h of co-incubation, resulting in a population distinct from the CAR^lo^/CD25^−^ group, which maintained the original CAR expression level (Fig. 1b–d). This population bifurcation is observed in both CD4^+^ and CD8^+^ T cells (Fig. 1b and Additional file 2: Figure S2), is independent of the co-stimulatory signals present in the CAR (either CD28 or 4-1BB; Fig. 1b, c), and is not restricted to a particular antigen (either CD19 or CD20; Fig. 1c, d). We also observed the emergence of CAR^hi^ cells after stimulating CAR-T cells with CD3/CD28 beads (Fig. 1e), indicating that the population bifurcation is not unique to antigen presentation by K562 cells or to the CAR signaling domain. Instead, it also occurs when T cells are stimulated via the endogenous T-cell receptor (TCR)/CD28 machinery. In all instances studied, CAR^lo^ cells exhibited low or no expression of the activation marker CD25 while CAR^hi^ cells showed robust CD25 upregulation (Fig. 1b–e), suggesting distinct activation states for the CAR^hi^ and CAR^lo^ populations.

### CAR^hi^ but not CAR^lo^ cells exhibit robust T-cell functions

**Fig. 2:**
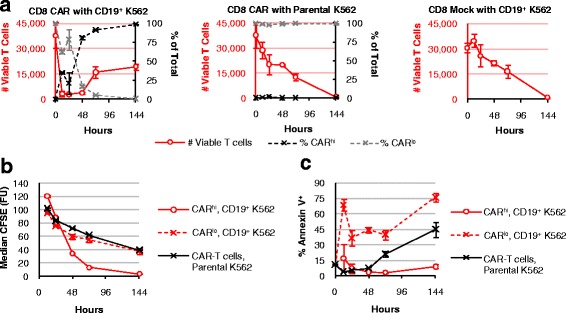
Antigen-stimulated CAR-T cells undergo selective expansion of the CAR^hi^ compartment. CFSE-labeled, CD8^+^ CAR-expressing cells or mock-transduced (CAR^−^) T cells were co-incubated with parental (CD19^−^) or CD19^+^ K562 targets without exogenous cytokines and monitored for **a** total T-cell count (left red axis) and CAR^hi^ or CAR^lo^ as a fraction of total CAR-T cells (right black axis), **b** median CFSE intensity, and **c** Annexin V staining. Average values of triplicates are shown with error bars indicating ± 1 standard deviation (s.d.)

Given their differences in CD25 expression, we hypothesized that the CAR^hi^ subpopulation is productively activated while CAR^lo^ cells remain inactive or are anergized. During co-incubation with CD19^+^ target cells in the absence of exogenous cytokines, CD8^+^ T cells expressing the CD19 CAR undergo a rapid and dramatic initial decline in viable T-cell count, followed by a population rebound in which CAR^hi^ cells emerge as the dominant T-cell group (Fig. 2a), consistent with the hypothesis that only CAR^hi^ cells are productively activated. In contrast, mock-transduced T cells as well as CAR-T cells co-incubated with parental (CD19^−^) K562 cells show a gradual decline in viable T-cell count without changes in CAR expression level, indicating that the dynamic population changes observed in Fig. 2a are specifically triggered by antigen stimulation. Primary CD4^+^ CAR-T cells as well as mixed CD4^+^ and CD8^+^ CAR-T cells show the same patterns of population dynamics (Additional file 3: Figure S3), demonstrating that the behavior is not unique to the CD8^+^ phenotype and is also representative of mixed CD4^+^ and CD8^+^ T cells typically used in therapeutic settings.

CFSE dilution and Annexin V staining results demonstrate that CAR^hi^ cells proliferate robustly with minimal apoptosis; in contrast, CAR^lo^ cells divide at the same low rate as unstimulated CAR-T cells, and exhibit increased cell death compared to both CAR^hi^ and unstimulated cells (Fig. 2b, c). These results further support the hypothesis that the CAR^lo^ cells, despite expressing a second-generation CAR containing a co-stimulation domain, have not been properly activated.

**Fig. 3:**
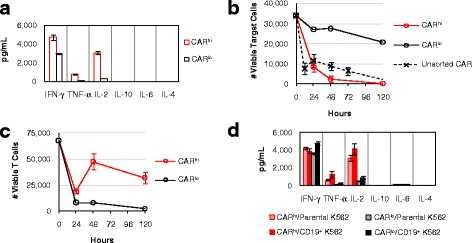
CAR^hi^ and CAR^lo^ T cells exhibit distinct functional capabilities. **a** CAR^hi^ and CAR^lo^ cells were sorted and subsequently cultured without antigen stimulation or exogenous cytokines. Cytokine production was measured 24 h post sorting. **b-d** Sorted CAR^hi^ and CAR^lo^ cells were co-incubated with CD19^+^ K562 targets without exogenous cytokines and monitored for **b** viable target-cell count, **c** viable T-cell count, and **d** cytokine production after 24 h of co-incubation. Sorted CAR^lo^ values in **b** and **c** are from single samples due to the rarity of viable cells recovered for this population from cell sorting. For all other samples, average values of triplicates are shown with error bars indicating ± 1 s.d.

In addition to differences in proliferative potentials, CAR^hi^ cells exhibit clearly superior functional profiles compared to CAR^lo^ cells. The two CD19 CAR-T cell populations were separated by FACS after 20 h of co-incubation with CD19^+^ K562 target cells. Multiplex cytokine measurements revealed robust Th1 cytokine production by CAR^hi^ cells during the 24-h period following cell sorting (Fig. 3a). In contrast, CAR^lo^ cells produced relatively high levels of IFN-γ but not TNF-α or IL-2, consistent with previous study reporting the ability of anergic T cells to produce IFN-γ but not IL-2 [21] (Fig. 3a).

CAR-T cells have been reported to serve as serial killers of tumor cells in successfully treated patients [4], and the ability to maintain functionality in the face of high tumor burden and repeated stimulation is critical to the therapeutic efficacy of CAR-T cells. Upon re-exposure to target cells, sorted CD8^+^ CAR^lo^ T cells showed minimal target-cell lysis (Fig. 3b). In contrast, CD8^+^ CAR^hi^ T cells rapidly eliminated CD19^+^ K562 targets, achieving even more complete target clearance than unsorted CAR-T cells (Fig. 3b). Both T-cell populations underwent a contraction in viable T-cell count upon antigen exposure, but CAR^hi^ cells greatly outperformed CAR^lo^ cells in subsequent proliferation (Fig. 3c). Furthermore, CAR^hi^ but not CAR^lo^ cells robustly secreted the Th1 cytokines TNF-α and IL-2 upon antigen re-challenge (Fig. 3d). As previously observed, CAR^lo^ cells retained the ability to produce IFN-γ (Fig. 3d). The contrasts between CAR^hi^ and CAR^lo^ cells described above were also observed among CD4^+^ CAR-T cells (Additional file 4: Figure S4), indicating the functional disparities observed are intrinsic to CAR^hi^ vs. CAR^lo^ subpopulations and are not restricted to the CD8^+^ phenotype. The clear functional superiority of CAR^hi^ cells suggests that maximization of CAR^hi^ cells within a given CAR-T cell preparation may enhance the anti-tumor potential of the cell product. We next explored the source of the CAR^hi^ phenotype and methods to direct the T cell population toward this functional subset.

### The CAR^hi^ phenotype is a transient state of activation induced by antigen stimulation

**Fig. 4:**
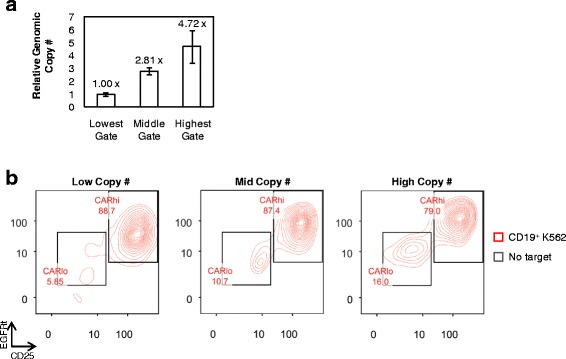
CAR^hi^ phenotype does not require high genomic copy number of CAR transgene. **a** Relative genomic CAR copy number in three sorted CAR–T-cell populations as determined by quantitative PCR relative to the housekeeping gene β-actin. The lowest copy number is set to 1. Values are averages of quadruplicates with error bars indicating ± 1 s.d. **b** CAR–T-cell populations with varying genomic CAR copy number separate into CAR^hi^/CD25^+^ and CAR^lo^/CD25^−^ populations within 18 h of antigen simulation

Efforts to maximize CAR^hi^ cells require knowledge of their origin. One possibility is that CAR^hi^ cells are genetically encoded with higher CAR copy numbers, and their superior proliferative capability enables their rapid enrichment after antigen stimulation, despite their apparent absence from the original CAR^+^ T-cell population. Alternatively, CAR^hi^ cells may be genetically similar to CAR^lo^ cells, but productive stimulation results in a distinct, activated state. To distinguish between these two possibilities, CD8^+^ T cells were sorted into three populations with different CAR expression levels prior to antigen stimulation (Additional file 5: Figure S5). After cell expansion over 9 days in culture, qPCR performed on genomic DNA confirmed significant differences in the copy number of the CAR transgene in the three populations (Fig. 4a). Upon antigen stimulation, all three populations were able to generate CAR^hi^ cells with clearly elevated CAR expression (Fig. 4b). In fact, T cells with the lowest CAR genomic copy number yielded the highest % CAR^hi^ and the highest CD25 expression level among CAR^hi^ cells upon antigen stimulation (Fig. 4b), indicating that a high genetic copy number of the CAR transgene is neither essential nor automatically conducive to the emergence of the CAR^hi^ phenotype.

**Fig. 5:**
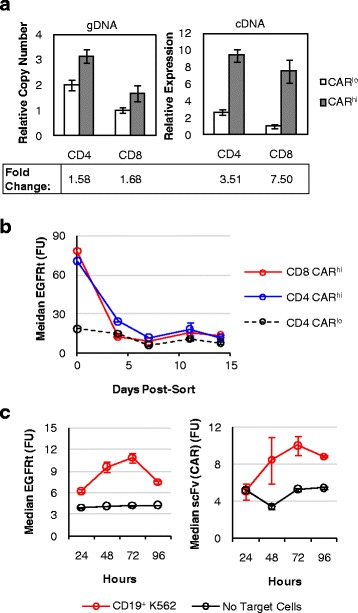
CAR^hi^ T cells arise from transient CAR transcript upregulation. **a** Genomic copy number and mRNA expression of CAR^hi^ and CAR^lo^ populations as determined by quantitative PCR relative to the housekeeping gene β-actin. The lowest expression level or copy number is set to 1. **b** EGFRt expression level of sorted cells maintained in culture with exogenous IL-2 and IL-15 and no antigen stimulation. **c** EGFRt and CAR surface expression on CD8^+^ CAR-T cells as measured by Erbitux and Protein-L staining, respectively, over 4 days of co-incubation with or without CD19^+^ K562 target cells. Values in **b** are averages of quadruplicates with error bars indicating ± 1 s.d.; all other plots show average values of triplicates with error bars indicating ± 1 s.d.

Quantitative PCR performed on sorted CAR^hi^ and CAR^lo^ cells showed that CAR^hi^ and CAR^lo^ cells differ slightly in genomic copy numbers of the CAR construct (Fig. 5a). However, the two populations diverge more prominently in CAR transcription levels, with CAR^hi^ cells overexpressing the anti-CD19 CAR mRNA by 3.5 folds (CD4^+^ T cells) or 7.5 folds (CD8+ T cells) compared to CAR^lo^ cells (Fig. 5a). In addition, CAR expression levels in CAR^hi^ cells return to baseline (i.e., the same level as unstimulated and CAR^lo^ cells) within days after the removal of antigen stimulation (Fig. 5b), indicating that the CAR^hi^ phenotype is predominantly due to transient upregulation of CAR expression. Furthermore, antigen-stimulated CAR-T cells upregulate the surface expression of both CAR and EGFRt, which are co-transcribed as one mRNA but translated into two separate proteins (Fig. 5c and Additional file 1: Figure S1D). This result indicates that the increase in CAR surface expression is not due to CAR-specific changes in post-translational processes such as surface localization, receptor endocytosis, or protein degradation. Taken together, these data indicate that elevated CAR expression in CAR^hi^ cells is mainly a result of transient CAR transcript upregulation in response to cell activation, and that increasing the copy number of the CAR transgene is unlikely to be the most effective means of promoting the CAR^hi^ phenotype.

### Overstimulation results in loss of the CAR^hi^ phenotype

**Fig. 6:**
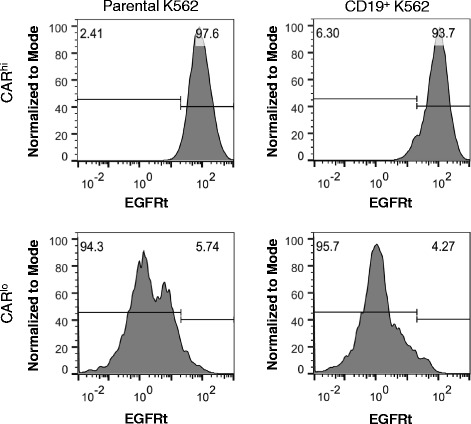
Antigen re-challenge results in CAR expression reduction. Sorted CD8^+^ CAR^hi^ and CAR^lo^ cells were assayed for EGFRt surface expression after 24 h of co-incubation. A small fraction of CAR^hi^ cells becomes CAR^lo^ upon antigen re-challenge, while a significant reduction in CAR expression is observed among CAR^lo^ cells after stimulation

One approach to maximizing CAR^hi^ cells is to prevent the dysfunctional CAR^lo^ state. Given that the CAR^hi^ phenotype is induced by antigen stimulation, we next investigated whether the CAR^lo^ cells fail to upregulate CAR expression because they experienced inadequate stimulation or if they became exhausted due to overstimulation. When sorted CAR^hi^ cells were re-challenged with antigen, the majority retained elevated CAR expression but a small population fell into the CAR^lo^ gate (Fig. 6), suggesting that repeated antigen exposure anergized a minority of the CAR^hi^ cells. Meanwhile, a large portion of CAR^lo^ cells experienced further reduction in CAR expression and no cells moved into the CAR^hi^ regime upon antigen re-challenge (Fig. 6). These results support the hypothesis that a sufficiently strong stimulation is required for the CAR^hi^ phenotype, but overstimulation results in the exhaustion of activated cells and a decline into the dysfunctional CAR^lo^/CD25^−^ state. Additional antigen challenge to CAR^lo^ cells would only further decrease CAR expression and T-cell functionality. These results are consistent with our previous observation that T cells with the highest genomic copy number of the CAR transgene (and thus the highest capacity to receive antigen stimulation) yielded the largest proportion of CAR^lo^ cells after antigen stimulation (Fig. 4b). Therefore, maximization of the CAR^hi^ population requires precise calibration of antigen stimulation, and a systematic approach to this task would facilitate the optimization of the cell production process.

### Persistent antigen stimulation facilitates CAR^hi^ expansion

Several stimulation conditions were evaluated to determine an effective protocol for the preparation of T cells with the CAR^hi^ phenotype. Since co-stimulation plays a major role in achieving productive T-cell activation, we first examined whether additional co-stimulation could enhance CAR^hi^ development and forestall the emergence of non-functional CAR^lo^ cells. However, supplementing a CD28 agonist antibody to CAR-T cells immediately prior to antigen exposure did not significantly alter the CAR^hi^ vs. CAR^lo^ distribution dynamics nor impact the absolute CAR^hi^ cell numbers (Additional file 6: Figure S6).

**Fig. 7:**
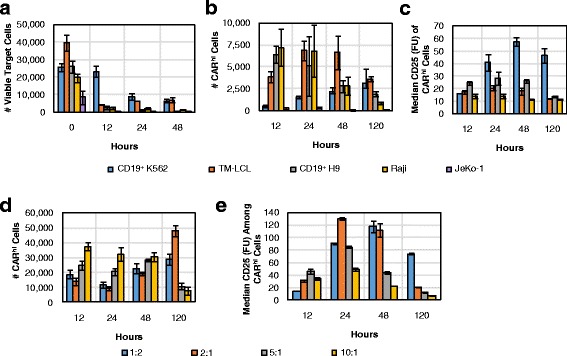
Varying CAR-T cell stimulation conditions impacts the dynamics of the CAR^hi^ response. **a-c** 1 × 10^5^ CD8^+^ CAR-T–cells were co-incubated with various target cell lines at 2:1 E:T ratio and monitored for **a** target cell counts, **b** number of CAR^hi^ T cells, and **c** CD25 expression among CAR^hi^ cells. **d-e** 2.5 × 10^5^ CD8^+^ CAR-T cells were co-incubated with CD19^+^ K562 at various E:T ratios and monitored for **d** number of CAR^hi^ cells and **e** CD25 expression among CAR^hi^ cells. Average values of triplicates are shown with error bars indicating ± 1 s.d.

T-cell expansion with the aid of antigen-presenting feeder cells is a well-established method for CAR-T cell preparation [18, 22, 23]. We next evaluated multiple feeder cell lines for their ability to support CAR^hi^/CD25^+^ cell generation (Fig. 7a–c). CD8^+^ CAR-T cells were co-incubated at a 2:1 effector-to-target (E:T) ratio with several CD19^+^ target cell lines, including naturally CD19^+^ JeKo-1, Raji, and TM-LCL cells, as well as K562 and H9 cells modified to stably express CD19. Besides JeKo-1 cells, which were depleted rapidly, all target cell lines tested resulted in the emergence of CAR^hi^ cells, and CAR^hi^ expression was always correlated with CD25 upregulation (Additional file 7: Figure S7). However, not all CAR^hi^ cells upregulated the activation marker equally (Fig. 7c). Considering both the number of CAR^hi^/CD25^+^ cells and the intensity of CD25 expression (Fig. 7b, c), the results suggest CD19^+^ K562 and TM-LCL cells are both suitable candidates as feeder cells for CD19 CAR-T cell expansion. Interestingly, these two cell lines are also the most resistant to CAR-T cell–mediated lysis among the target cell lines tested (Fig. 7a). In contrast, antigen expression level on target cells shows no correlation with the target cells’ susceptibility to T-cell–mediated lysis or with CAR^hi^ cell development (Additional file 8: Figure S8). Therefore, it is the persistence of antigen presentation rather than the antigen density on individual target cells that predicts CAR^hi^ emergence patterns. The most resilient target cell line, CD19^+^ K562, resulted in a relatively small CAR^hi^ population at early time points, but the total number and CD25 expression level of CAR^hi^ cells increased steadily throughout subsequent days, confirming CD19^+^ K562 as an effective trigger for the functional CAR^hi^ phenotype (Fig. 7a—c).

To more precisely evaluate the impact of target-cell dosage on CAR–T-cell bifurcation, we co-incubated CD8^+^ CAR-T cells with CD19^+^ K562 targets at 1:2, 2:1, 5:1, and 10:1 E:T ratios. Results corroborate the observation that sustained production of CAR^hi^ cells requires high target-cell inputs that enable persistent antigen presentation (Fig. 7d). CAR-T cells treated with the largest number of antigen-presenting cells ultimately resulted in the highest median CD25 expression level among CAR^hi^ cells, but only after a steady rise over time as previously observed (Fig. 7e). These observations hold true regardless of whether the target cells were irradiated prior to co-incubation with CAR-T cells (Additional file 9: Figure S9), confirming the applicability of this evaluation method to CAR-T cell expansion protocols employed in clinical settings.

### CAR^hi^ cells are PD-1^+^ but resist PD-L1-induced dysfunction

**Fig. 8:**
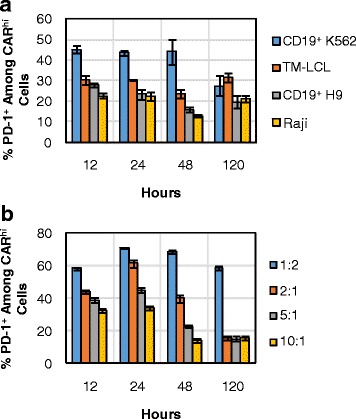
PD-1 is upregulated in CAR^hi^ cells with intensities dependent upon stimulation conditions. CD8^+^ CAR-T cells were co-incubated with **a** various target cell lines or **b** CD19^+^ K562 target cells at various E:T ratios. % PD-1^+^ among CAR^hi^ cells was determined by surface antibody staining. Average values of triplicates are shown with error bars indicating ± 1 s.d.

The observation that sustained antigen stimulation is required for the maintenance of the CAR^hi^ phenotype (Figs. 5b and 7b, d) raises the question of whether CAR^hi^ cells are at risk of exhaustion, leading to lower therapeutic efficacy despite their functional capabilities *in vitro*. Indeed, surface antibody staining revealed that CAR^hi^ cells upregulate PD-1, a marker whose sustained expression is generally associated with T-cell dysfunction [24] (Fig. 8). Furthermore, CAR^hi^ cells generated through stimulation by the most resilient antigen-presenting target cells (CD19^+^ K562) and the highest antigen concentration (1:2 E:T ratio) also express the highest levels of PD-1 (Fig. 8). Although it is unsurprising that antigen-stimulated cells upregulate PD-1, our target-cell lysis, T-cell proliferation, and cytokine production assay results did not reveal any sign of dysfunction among the PD-1^+^ CAR^hi^ cells (Fig. 3), contrary to previous reports on PD-1^+^ tumor-targeting T cells [25–28]. One possible explanation is that the target cell line used in this study (K562) did not express high levels of PD-L1 (Additional file 10: Figure S10A). To investigate this possibility, the CD19^+^ K562 target line was engineered to stably overexpress PD-L1 (Additional file 10: Figure S10) and used in weeklong co-incubation assays with both CD4^+^ and CD8^+^ CD19 CAR-T cells.

**Fig. 9:**
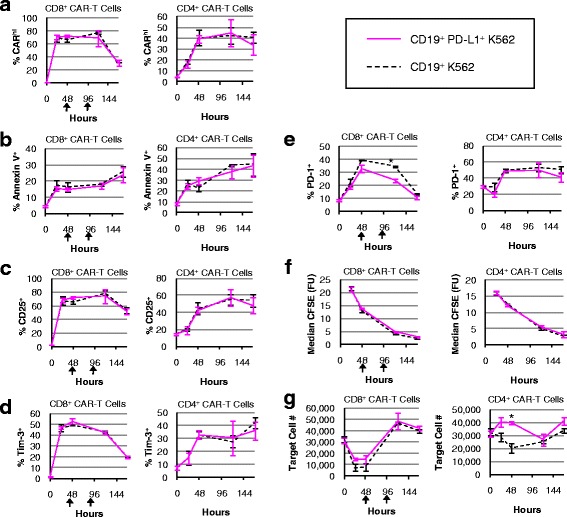
CAR-T cells are minimally impacted by PD-L1 expression on target cells. CD8^+^ and CD4^+^ CAR-T cells were co-incubated with CD19^+^ or CD19^+^/PDL1^+^ K562 target cells without exogenous cytokines and monitored for **a** % CAR^hi^, **b** % Annexin V^+^, **c** % CD25^+^, **d** % Tim-3^+^, **e** % PD-1^+^, **f** CFSE intensity, and **g** target cell count. An asterisk indicates significant differences comparing co-incubations with CD19^+^ versus CD19^+^/PDL1^+^ K562 target cells at that time-point as determined by two-tailed unpaired Student’s *t* tests with the Bonferroni correction (*p* = 0.00019 in **e** and *p* = 0.00076 in **g**). Black arrows denote the addition of target cells to ensure sustained exposure to PD-L1. Average values of triplicates are shown with error bars indicating ± 1 s.d.

CD8^+^ CAR-T cells monitored over seven days of co-incubation in the absence of exogenous cytokines showed no susceptibility to high PD-L1 expression on target cells, demonstrating little to no changes in % CAR^hi^, % Annexin V^+^, CD25 and Tim-3 expression, T-cell proliferation, and target-cell lysis efficiency (Fig. 9). A small but statistically significant reduction in PD-1 expression was observed in CD8^+^ CAR-T cells upon co-incubation with PD-L1^+^ target cells (Fig. 9e). CAR^hi^ expression was consistently correlated with increased CD25, PD-1, and Tim-3 expression (Additional file 11: Figure S11), but high levels of PD-L1 on target cells did not result in reduced proliferative or target-cell lysis response by CD8^+^ CAR-T cells over the 7-day observation period (Fig. 9f, g). It should be noted that since CD8^+^ CAR-T cells are capable of eliminating CD19^+^ K562 target cells, additional target cells were added to the co-incubation culture at 48 and 96 h to ensure sustained exposure of CAR-T cells to PD-L1 presentation by target cells. This continuous PD-L1 presentation did not result in noticeable impact on CD8^+^ CAR-T cell function. However, the repeated antigen challenge did result in a decline in CAR^hi^ cells relative to CAR^lo^ cells at 144 h (Fig. 9a), consistent with our previous observation that overstimulation contributes to the loss of the functional CAR^hi^ population (Fig. 6).

PD-L1 expression protected target cells from CD4^+^ CAR–T-cell lysis at early time points (Fig. 9g), potentially due to the higher PD-1 expression level in CD4^+^ cells compared to CD8^+^ cells (Additional file 12: Figure S12). However, the resistance to CD4^+^ T-cell–mediated lysis exhibited by PD-L1^+^ target cells appeared to be temporary, and PD-L1 expression on target cells did not impact any of the other T-cell parameters quantified (Fig. 9). No target cells beyond the initial input were added to the CD4^+^ culture since CD4+ CAR-T cells did not eliminate the original target population. Taken together, the results indicate that CAR^hi^ cells persistently upregulate PD-1 expression but are not functionally impaired by PD-L1 presentation on K562 target cells.

### CAR-T cells generated from T_CM_ subset are primed for the CAR^hi^/CD25^+^ phenotype

**Fig. 10:**
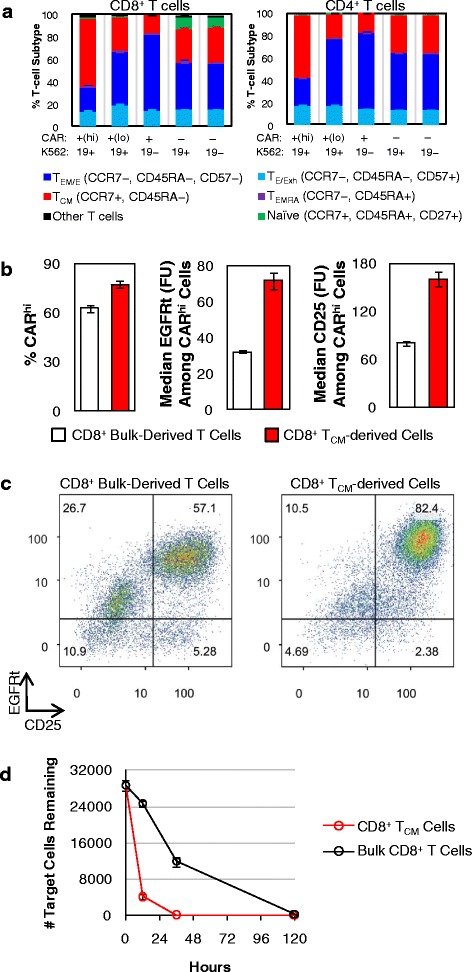
T_CM_-derived CAR-T cells produce more CAR^hi^ T cells. **a** Bulk T-cell–derived CD4^+^ and CD8^+^ CAR-T cells were co-incubated with CD19^+^ K562 target cells without exogenous cytokines, and T-cell subtype distribution was quantified after 24 h. T_CM_: central memory T cells; T_EM_: effector memory T cells; T_EMRA_: effector memory-CD45RA^+^ T cells; T_E_: effector T cells; T_Exh_: exhausted T cells. **b** Bulk- and T_CM_-derived CD8^+^ CAR-T cells were co-incubated with CD19^+^ K562 target cells, and the % CAR^hi^ and EGFRt and CD25 expression levels among CAR^hi^ cells were quantified. **c** Both the bulk- and T_CM_-derived populations separated into CAR^hi^/CD25^+^ and CAR^lo^/CD25^−^, with the T_CM_-derived sample showing higher CD25 expression overall. In both samples, a subpopulation of contaminating CAR^−^ cells was also CD25^+^, possibly due to paracrine stimulation from CAR^+^ cells. **d** Target cell lysis was monitored over 5 days. Average values of triplicates are shown with error bars indicating ± 1 s.d.

As demonstrated above, antigen stimulation is an integral part of CAR-T cell preparation and its calibration can significantly influence the efficiency of CAR^hi^ generation. Another important parameter in CAR-T cell preparation is the specific subtype of T cells used to make CAR-T cells. Surface antibody staining revealed that the CAR^hi^ population is enriched in central memory T (T_CM_) cells while the CAR^lo^ population consists of predominantly effector (T_E_) and effector memory T (T_EM_) cells (Fig. 10a). We thus investigated whether enrichment for T_CM_ cells prior to CAR transgene transduction may increase the CAR-T cells’ potential to attain the CAR^hi^ phenotype.

From the same healthy donor’s blood sample, bulk CD8^+^ T cells and CD8^+^ T_CM_ (CCR7^+^/CD45RA^−^) cells were isolated separately (Additional file 13: Figure S13A), stimulated with CD3/CD28 beads, lentivirally transduced with CD19 CAR transgene, and sorted for CAR^+^ expression (Additional file 13: Figure S13B). Without further expansion, sorted CAR^+^ cells were co-incubated with CD19^+^ K562 target cells for 36 h. CAR^hi^/CAR^lo^ bifurcation was observed in both T-cell populations, but the T_CM_-derived population yielded more CAR^hi^ cells, and its CAR^hi^ cells had higher CAR and CD25 expression levels (Fig. 10b). Consistent with all previous experiments, which were performed using bulk CD8+ T cells, CAR^lo^ cells from the bulk CD8^+^-derived CAR-T sample were CD25^−^. In contrast, CAR^lo^ cells from the T_CM_-derived sample had partially upregulated CD25 (Fig. 10c), indicating that the T_CM_-derived cells are both primed for the CAR^hi^ phenotype and able to retain activation status even when CAR expression levels are low. This conclusion is further supported by the observation that the T_CM_-derived cells were much more effective at lysing the target cells (Fig. 10d).

We monitored the T_CM_ status of the two populations throughout the cell preparation process as well as after antigen stimulation. Surface staining of CCR7 and CD45RA revealed that most cells in the T_CM_-enriched population had lost CCR7 expression by the time of cell sorting for CAR expression (Additional file 14: Figure S14A), a progression that is consistent with previous reports of *ex vivo* T_CM_ cell expansion [15, 16]. However, antigen stimulation caused a re-enrichment of the CCR7^+^/CD45RA^−^ phenotype, particularly among CAR^hi^ cells (Additional file 14: Figure S14B). In contrast, the bulk CD8^+^ T cell population enriched for CCR7 expression through the course of CD3/CD28 bead stimulation, and further increased CCR7 expression upon antigen stimulation (Additional file 14: Figure S14CD). Taken together, these results indicate the use of T_CM_ cells as the starting population is conducive to the production of functionally superior CAR-T cells, even though the dominant phenotype remains fluid throughout the course of T-cell expansion and antigen stimulation.

## Discussion

Recent successes in CD19 CAR–T-cell trials have demonstrated the remarkable therapeutic potential of CD19 CAR-T cells and fueled intense interest in the development of CAR–T-cell therapies against additional tumor targets. As CAR–T-cell technology moves beyond experimental status, it is critical that effective cell-manufacturing protocols and characterization methods are developed to ensure robust and reproducible generation of CAR-T cells. The various clinical trials completed thus far have employed different T-cell manufacturing protocols unique to each research group, and the relative merits of each have been judged based on overall clinical outcome rather than a detailed examination of differences in T-cell characteristics. It is also difficult to elucidate whether any particular step in the preparation process may have affected the phenotype and functionality of the resulting CAR-T cells. As a result, efforts to improve cell preparation protocols remain empirically driven, with few guiding cues on which parameters to modify.

Here, we presented a systematic study on the phenotypic and functional changes of CAR-T cells after T-cell activation. We demonstrated that activated T cells show a clear pattern of population bifurcation into CAR^hi^/CD25^+^ vs. CAR^lo^/CD25^−^ groups. This bifurcation appears to be general to T cells activated *in vitro*, as it is observed in both CD4^+^ and CD8^+^ T cells, in T cells stimulated through either TCRs or CARs targeting different antigens, and in T cells expressing CARs that contain either CD28 or 4-1BB co-stimulatory signals. The transient increase of CAR expression upon antigen stimulation has been observed in previous studies [29, 30], but to our knowledge, no detailed characterization of the difference between CAR^hi^ and CAR^lo^ cells has been performed. In this study, we discovered that CAR^hi^ cells consistently upregulate expression of the activation marker CD25, and target-cell lysis, cytokine production, and T-cell proliferation assays demonstrate that CAR^hi^/CD25^+^ cells are functionally superior to their CAR^lo^/CD25^−^ counterparts. In fact, CAR^lo^/CD25^−^ cells show multiple signs of anergy and are unable to execute anti-tumor functions. This observation is of practical importance because the CAR^lo^/CD25^−^ population is CAR^+^ with similar CAR genomic copy numbers as CAR^hi^ cells; thus, they would have satisfied the release criteria typically applied in CAR–T-cell trials [4, 31] despite their lack of effector functions. The ability to distinguish and characterize this population was contingent upon the detailed *in vitro* examination of T-cell phenotype changes post stimulation.

Our results show that the CAR^hi^/CD25^+^ phenotype is a transient response to antigen stimulation rather than a genetically hard-wired population destined for superior function. Past studies have demonstrated that T-cell activation can temporarily enhance transgene expression from constitutive promoters, but have not revealed the underlying mechanisms [32, 33]. A scan of the EF1α promoter used in the current study and in multiple clinical trials [31, 34, 35] reveals binding sequences for TFII-I and Sp1, both widely employed transcription factors. In particular, TFII-I has been shown to be rapidly phosphorylated upon CD3 crosslinking and upregulated in activated CD4^+^ T cells [36, 37]. It is possible that T-cell signaling increases the level of these transcription factors and results in the upregulation of CAR expression from the EF1α promoter. Further investigations are necessary to conclusively elucidate the mechanism of transient CAR upregulation upon antigen stimulation.

Although CAR^hi^/CD25^+^ cells eventually return to a CAR^lo^/CD25^−^ phenotype after antigen removal, they mount a robust cytolytic and cytokine-production response when re-challenged with antigen-expressing targets. This is in stark contrast to CAR^lo^/CD25^−^ cells, which remain non-functional upon re-exposure to antigen. Therefore, the ability to bias CAR-T cells toward the CAR^hi^/CD25^+^ phenotype during the cell-preparation stage has the potential to increase the therapeutic capability of T cells against targeted tumor cells. We demonstrated that, for a second-generation CAR containing CD28 co-stimulatory domain, the application of extra co-stimulation via agonistic CD28 antibody does not alter the relative distribution of the CAR^hi^ vs. CAR^lo^ subpopulations, but sustained antigen stimulation shows strong correlations with CAR^hi^ cell generation. Interestingly, the absolute density of antigen on target cells did not correlate with the intensity of T-cell response or the effectiveness of CAR^hi^ cell generation. Among the antigen-presenting cells tested, CD19^+^ K562 and TM-LCL appear to present levels of antigen stimulation that are conducive to robust CAR^hi^/CD25^+^ cell production, consistent with TM-LCL’s successful use in clinical protocols [23, 38]. It remains possible that characteristics in addition to persistence, such as co-stimulatory signals present on the target cell surface, contribute to the effectiveness of CD19^+^ K562 and TM-LCL in supporting the generation of CAR^hi^ cells. Given the apparent sensitivity of CAR^hi^ cell production to the type and duration of antigen presentation, and the multiple degrees of variability that exist among potential feeder cell lines used in *ex vivo* T-cell expansion, the detailed *in vitro* characterization approach described in this study may be used to systematically fine-tune the antigen-presenting cell type and E:T ratio required for the efficient production of functionally superior CAR-T cells.

In addition to the type and degree of antigen stimulation used to expand CAR-T cells, the specific T-cell subtype used to generate CAR-T cells also greatly influences the efficiency of CAR^hi^ cell generation. Our study demonstrated that CAR^hi^ cells are enriched in the T_CM_ phenotype, and T_CM_-derived CAR-T cells are functionally superior to those made from bulk CD8^+^ T cells. These observations are consistent with the previously reported observation that T_CM_ cells are superior to T_EM_ cells in establishing long-term persistence in primates [15]. In this and most other CAR–T-cell characterization studies, *in vivo* results are viewed as the most relevant and credible proof of CAR function. Although the value of *in vivo* data is clear, many important features of CAR-T cell biology—particularly dynamic changes over time in phenotype and function—are impossible to obtain at high enough resolution *in vivo*. Features such as CAR^lo^ cells cannot be detected in animal models or patients because they are quickly depleted *in vivo*, but the knowledge of their existence and detailed *in vitro* characterizations of such populations provide valuable information on how to improve CAR-T cell production so as to maximize the number of cells that will persist and execute antitumor functions upon adoptive transfer.

Recent clinical studies have demonstrated the exciting potential of checkpoint inhibitor therapies such as CTLA-4 and PD-1 blockade, which boost T-cell responses by preventing T-cell exhaustion and anergy. Our observation that the CAR^hi^ phenotype is triggered by antigen stimulation and maintained by prolonged antigen exposure raised the question of whether CAR^hi^ cells may be susceptible to exhaustion or dysfunction. We indeed observed significant and sustained PD-1 upregulation among CAR^hi^ cells. However, no functional impairment was observed even when CAR^hi^ cells were challenged over multiple days with a constant supply of target cells that strongly overexpress PD-L1. It should be noted that the K562 target cells used in this study do not express the co-stimulatory molecules CD83, CD86, 4-1BB ligand, OX40 ligand, and ICOS ligand, and they express very low levels of CD80 [39]. Past studies have needed to engineer increased expression of these co-stimulatory molecules on K562s in order to prime and expand T cells [39–41]. Therefore, the K562 cells used in our study, which have not been engineered to express co-stimulatory molecules, are unlikely to significantly mitigate PD-1/PD-L1 signaling via CAR-independent co-stimulation. Our results contrast with reports of PD-L1 sensitivity exhibited by T cells stimulated via natural TCRs [42–44], and indicate an unexpected source of resilience in CAR-T cells against PD-1–mediated cell inactivation. Intensive T-cell stimulation with CD3 and CD28 antibodies has been found to overcome PD-1/PD-L1 inhibition of T-cell proliferation *in vitro* [45]; it is possible that robust expression and activation of the exogenous CAR containing a CD28 co-stimulatory domain similarly overwhelms PD-1 signaling. However, PD-L1 blockade has been reported to restore activity in hypofunctional CAR-T cells *ex vivo* after recovery from mouse tumors [46], suggesting the PD-1 pathway does play a role in maintaining the dysfunction of exhausted CAR-T cells. Since PD-1 blockade can indirectly impact CAR-T cell function (e.g., by reducing the prevalence of myeloid derived suppressor cells in the tumor microenvironment [47]), co-administration of CAR-T cell therapy with CTLA-4 or PD-1 blockade remains an intriguing therapeutic option. Nevertheless, our results suggest that PD-1 upregulation—even when sustained over days in the presence of PD-L1–expressing target cells—does not automatically relegate CAR-T cells to anergy or exhaustion.

## Conclusions

In this study, we have identified and characterized a pattern of population bifurcation that results in two distinct T-cell groups with dramatic differences in proliferative and anti-tumor capacities post antigen stimulation. The combination of CAR and CD25 expression levels reliably predicts the functionality of a given T-cell population, thus providing a convenient and robust set of phenotypic markers that can aid future efforts to optimize T-cell manufacturing protocols. Characterization results in our study indicate that starting with an enriched T_CM_ population and carefully tuning the persistence of antigen stimulation during the manufacturing stage may direct the T-cell population toward the functionally superior phenotype, and minimize a source of variability in T-cell quality that is largely undetectable through standard product-release testing and *in vivo* models. As adoptive T-cell therapy makes strides in clinical outcomes, it is increasingly important that robust and reproducible T-cell manufacturing protocols be optimized in a systematic manner. Additional in-depth characterization studies on the phenotypic changes experienced by T cells throughout the manufacturing and post-infusion period will yield valuable information and guide the continuing effort to improve adoptive T-cell therapy.

## Additional files

Additional file 1: Figure S1. EGFRt expression directly correlates with CAR expression. CD8^+^ T cells were transduced with a *cd19 car-t2a-egfrt* construct containing the CH2 and CH3 domains in the IgG4 spacer to allow for anti-Fc antibody binding. (A) Unsorted transduced cells, (B) sorted, resting CAR-T cells, (C) CAR-T cells co-incubated with parental K562 cells, and (D) CAR-T cells co-incubated with CD19^+^ K562 targets cells were co-stained with Erbitux and anti-Fc antibodies that bind to EGFRt and the CAR extracellular spacer, respectively. Additional file 2: Figure S2. CD4^+^ CAR-T cells polarize into CAR^hi^ and CAR^lo^ populations. CD4^+^ T cells bifurcate into CAR^hi^/CD25^+^ and CAR^lo^/CD25^−^ populations upon co-incubation with on-target K562 cells for 24 h. Additional file 3: Figure S3. Antigen-stimulated CD4^+^ as well as mixed CD4^+^ and CD8^+^ CAR-T cells demonstrate an initial population crash followed by selective expansion of CAR^hi^ cells. (A) CD4^+^ or (B) mixed CD4^+^ and CD8^+^ CAR-T cells were co-incubated with parental and CD19^+^ K562 targets without exogenous cytokines and monitored for total T-cell count (left axis) and CAR^hi^ or CAR^lo^ as a proportion of total CAR-T cells (right axis). Average values of triplicates are shown with error bars indicating ± 1 s.d. Additional file 4: Figure S4. CD4^+^ CAR^hi^ and CAR^lo^ T cells exhibit distinct functional capabilities. Sorted CD4^+^ CAR^hi^ and CAR^lo^ cells were co-incubated with CD19^+^ K562 targets without exogenous cytokines and monitored for (A) viable target-cell count, (B) viable T-cell count, and (C) cytokine production after 24 h of co-incubation. Sorted CAR^lo^ values are from single samples due to the rarity of viable cells recovered for this population from cell sorting. For all other samples, average values of triplicates are shown with error bars indicating ± 1 s.d. Additional file 5: Figure S5. Isolation of CAR-T cell populations with different CAR expression levels. Transduced, non–antigen-stimulated CD8^+^ T cells were sorted by FACS into three populations with different EGFRt expression levels (Low, Mid, and High). The y-axis is an irrelevant channel and cells were not stained with DAPI. Additional file 6: Figure S6. Additional CD28 co-stimulation does not impact the CAR^hi^ response. CD8^+^ CAR-T cells co-incubated with CD19^+^ K562 target cells at 2:1 E:T ratio with or without CD28 agonist were monitored for proportion and total number of CAR^hi^ cells. Average values of triplicates are shown with error bars indicating ± 1 s.d. The difference in the number of CAR^hi^ cells at 120 h was determined to be not significant (n.s.) by the two-tailed unpaired Student’s *t* test with the Bonferroni correction (*p* = 0.0376, which is greater than the 0.0125 significance level after the Bonferroni correction for multiple comparisons). Additional file 7: Figure S7. CAR and CD25 upregulation are directly correlated in antigen-stimulated T cells. CAR-T cells bifurcate into CAR^hi^/CD25^+^ and CAR^lo^/CD25^−^ subpopulations upon co-incubation with CD19^+^ K562, TM-LCL, CD19^+^ H9, and Raji target cells for 24 h. JeKo-1 cells were rapidly eliminated and did not elicit a CAR^hi^ response. Additional file 8: Figure S8. CD19 expression levels of various target cell lines. CD19^+^ H9, CD19^+^ K562, Raji, JeKo-1, TM-LCL, and parental K562 cell lines were stained with an anti-CD19 antibody or an isotype control. Numbers above each plot indicate the fold difference in median fluorescence intensity between CD19 and isotype control staining. Additional file 9: Figure S9. Stimulating CAR-T cells with varying amounts of irradiated CD19^+^ K562s impacts the dynamics of the CAR^hi^ response. (A) Number of CAR^hi^ and (B) median CD25 expression among CAR^hi^ cells in a co-incubation of CD8^+^ CAR-T cells with irradiated CD19^+^ K562 cells at various E:T ratios. Average values of triplicates are shown with error bars indicating ± 1 s.d. Additional file 10: Figure S10. PD-L1 can be overexpressed on otherwise PD-L1^−^ K562 target cells. (A) Various K562 target cell lines were stained with PD-L1 antibody. (B) K562 lines were also cross-stained for both PD-L1 and CD19 to verify antigen expression. Additional file 11: Figure S11. CAR^hi^ cells upregulate CD25, Tim-3, and PD-1 regardless of PD-L1 expression on target cells. CD8^+^ CAR-T cells were co-incubated with CD19^+^ or CD19^+^PD-L1^+^ target cells without exogenous cytokines and surface-stained with EGFRt, CD25, Tim-3, and PD-1 antibodies. Additional file 12: Figure S12. CD4^+^ CAR^hi^ T cells express higher levels of PD-1 than CD8^+^ CAR^hi^ T cells. CD4^+^ and CD8^+^ CAR-T cells were co-incubated with CD19^+^ target cells without exogenous cytokines and monitored by surface antibody staining for (A) % PD-1^+^ among CAR^hi^ cells and (B) median PD-1 fluorescence among PD-1^+^ cells. Average values of triplicates are shown with error bars indicating ± 1 s.d. Additional file 13: Figure S13. Isolation of T_CM_-derived CAR^+^ cells. (A) The T_CM_ subset (CD45RA^−^, CCR7^+^) was enriched from bulk CD8^+^ T cells. (B) Both T_CM_ and bulk CD8^+^ cells were transduced with CAR and sorted for the EGFRt^+^ fraction. Both positive (solid gray) and negative (dotted open) fractions were collected by magnetic bead sorting. Numbers shown indicate the frequency of EGFRt^+^ cells within the positive fraction. Additional file 14: Figure S14. Phenotypic shifts in the T_CM_- and bulk-CD8–derived cells. (A) CD45RA and CCR7 surface expression was monitored for T_CM_-derived cells prior to sorting for EGFRt^+^ cells, 4 days after sorting (immediately before co-incubation with target cells), and after 36 h of co-incubation with CD19^+^ target cells. (B) EGFRt and CCR7 surface expression after 36 h of co-incubation with CD19^+^ target cells. (C) and (D) show the corresponding data for bulk-CD8–derived cells.

## Abbreviations

CAR: Chimeric antigen receptor
E:T: Effector-to-target
EGFRt: Truncated epidermal growth factor receptor
scFv: Single-chain variable fragment
T2A: *Thosea asigna* virus 2A peptide
T_CM_: Central memory T cell
TCR: T-cell receptor
T_E_: Effector T cell
T_EM_: Effector memory T cell
