# Supplementary figures and images for: Identification and selective expansion of functionally superior T cells expressing chimeric antigen receptors
Source: J Transl Med. 2015 May 20;13:161. doi: 10.1186/s12967-015-0519-8 (PMC4457995; doi:10.1186/s12967-015-0519-8)

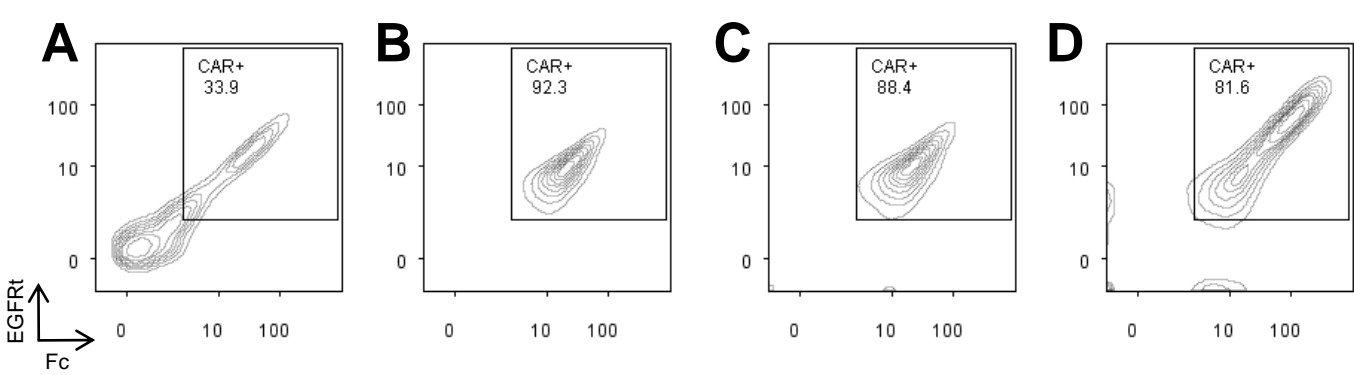

Supplement: Additional file 1: Figure S1. — EGFRt expression directly correlates with CAR expression. CD8+ T cells were transduced with a cd19 car-t2a-egfrt construct containing the CH2 and CH3 domains in the IgG4 spacer to allow for anti-Fc antibody binding. (A) Unsorted transduced cells, (B) sorted, resting CAR-T cells, (C) CAR-T cells co-incubated with parental K562 cells, and (D) CAR-T cells co-incubated with CD19+ K562 targets cells were co-stained with Erbitux and anti-Fc antibodies that bind to EGFRt and the CAR extracellular spacer, respectively. [file 12967_2015_519_MOESM1_ESM.pdf]

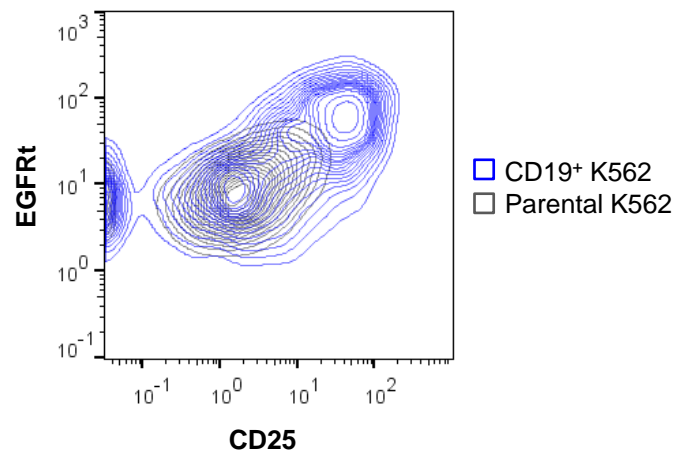

Supplement: Additional file 2: Figure S2. — CD4+ CAR-T cells polarize into CARhi and CARlo populations. CD4+ T cells bifurcate into CARhi/CD25+ and CARlo/CD25− populations upon co-incubation with on-target K562 cells for 24 h. [file 12967_2015_519_MOESM2_ESM.pdf]

**A**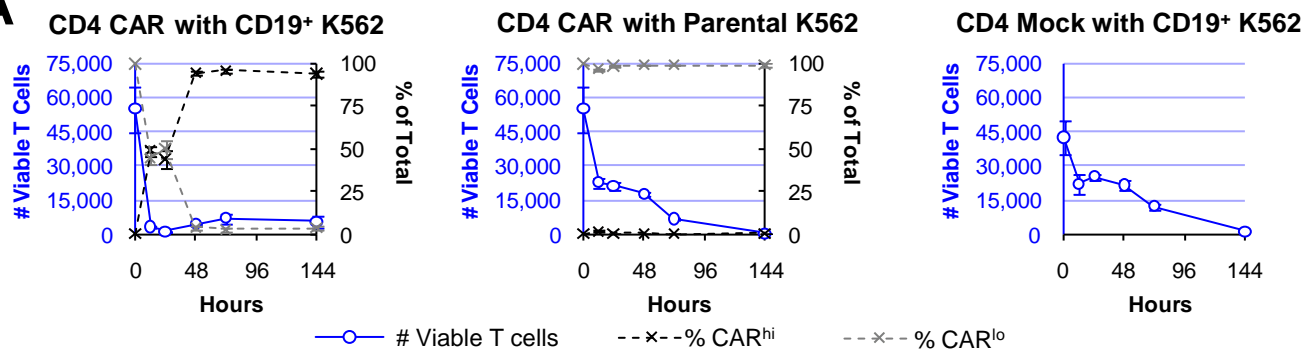**B**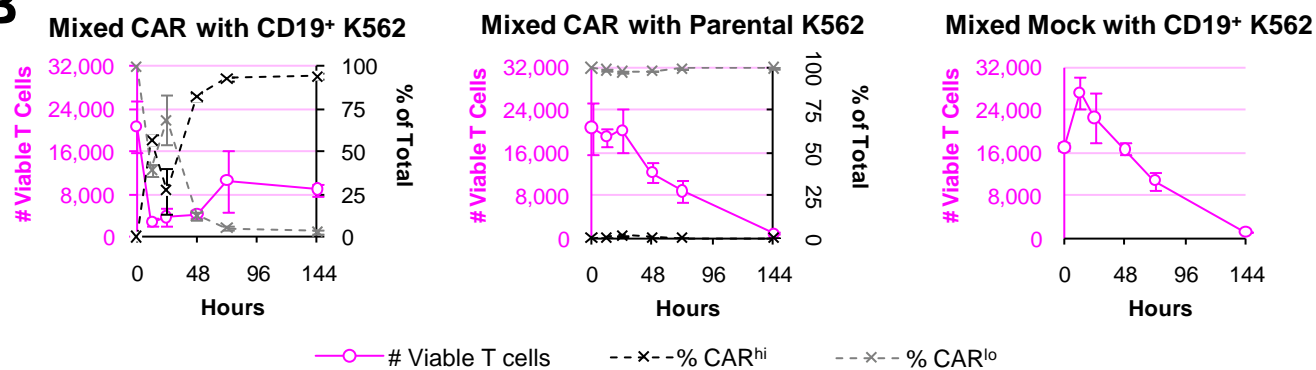

Supplement: Additional file 3: Figure S3. — Antigen-stimulated CD4+ as well as mixed CD4+ and CD8+ CAR-T cells demonstrate an initial population crash followed by selective expansion of CARhi cells. (A) CD4+ or (B) mixed CD4+ and CD8+ CAR-T cells were co-incubated with parental and CD19+ K562 targets without exogenous cytokines and monitored for total T-cell count (left axis) and CARhi or CARlo as a proportion of total CAR-T cells (right axis). Average values of triplicates are shown with error bars indicating ± 1 s.d. [file 12967_2015_519_MOESM3_ESM.pdf]

**A**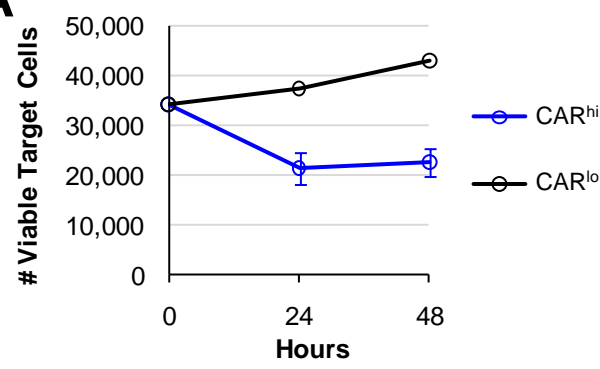**B**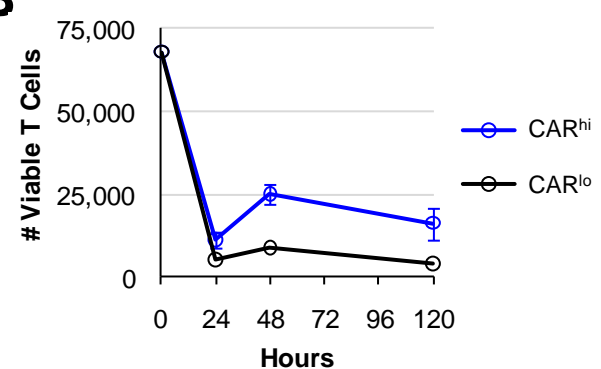**C**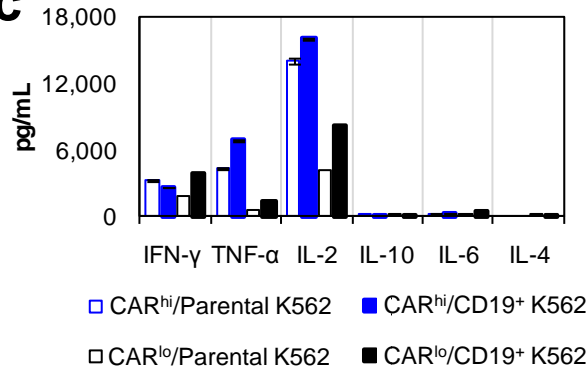

Supplement: Additional file 4: Figure S4. — CD4+ CARhi and CARlo T cells exhibit distinct functional capabilities. Sorted CD4+ CARhi and CARlo cells were co-incubated with CD19+ K562 targets without exogenous cytokines and monitored for (A) viable target-cell count, (B) viable T-cell count, and (C) cytokine production after 24 h of co-incubation. Sorted CARlo values are from single samples due to the rarity of viable cells recovered for this population from cell sorting. For all other samples, average values of triplicates are shown with error bars indicating ± 1 s.d. [file 12967_2015_519_MOESM4_ESM.pdf]

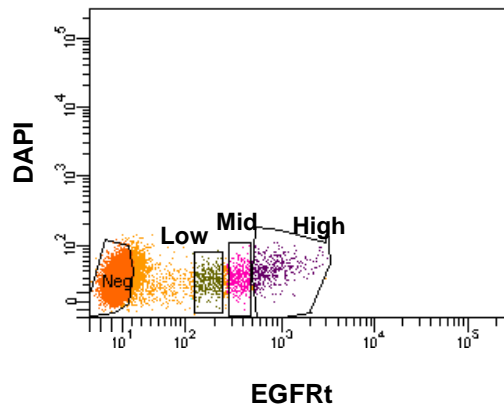

Supplement: Additional file 5: Figure S5. — Isolation of CAR-T cell populations with different CAR expression levels. Transduced, non–antigen-stimulated CD8+ T cells were sorted by FACS into three populations with different EGFRt expression levels (Low, Mid, and High). The y-axis is an irrelevant channel and cells were not stained with DAPI. [file 12967_2015_519_MOESM5_ESM.pdf]

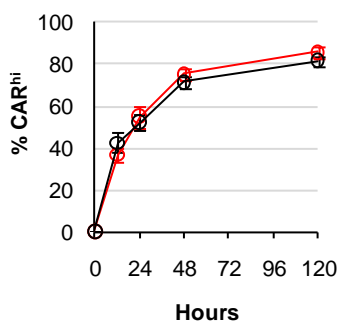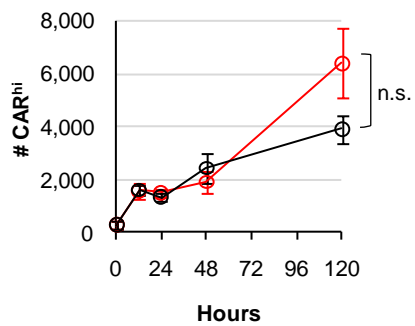

—○— CD19<sup>+</sup> K562 + anti-CD28

—○— CD19<sup>+</sup> K562

Supplement: Additional file 6: Figure S6. — Additional CD28 co-stimulation does not impact the CARhi response. CD8+ CAR-T cells co-incubated with CD19+ K562 target cells at 2:1 E:T ratio with or without CD28 agonist were monitored for proportion and total number of CARhi cells. Average values of triplicates are shown with error bars indicating ± 1 s.d. The difference in the number of CARhi cells at 120 h was determined to be not significant (n.s.) by the two-tailed unpaired Student’s t test with the Bonferroni correction (p = 0.0376, which is greater than the 0.0125 significance level after the Bonferroni correction for multiple comparisons). [file 12967_2015_519_MOESM6_ESM.pdf]

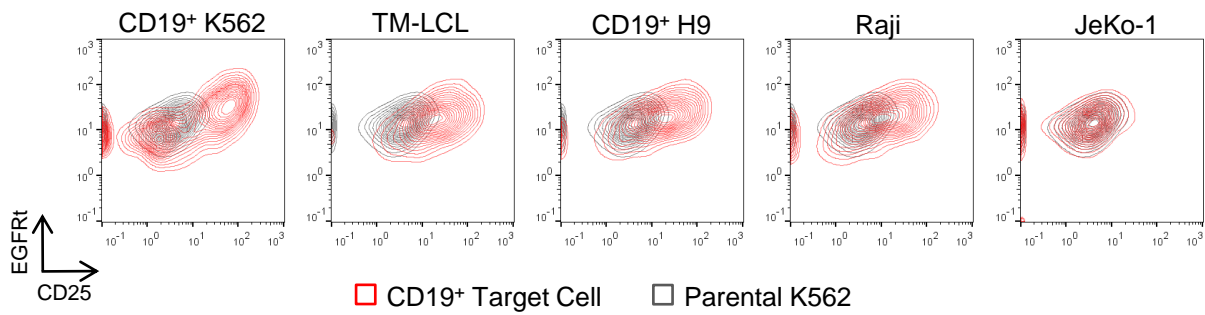

Supplement: Additional file 7: Figure S7. — CAR and CD25 upregulation are directly correlated in antigen-stimulated T cells. CAR-T cells bifurcate into CARhi/CD25+ and CARlo/CD25− subpopulations upon co-incubation with CD19+ K562, TM-LCL, CD19+ H9, and Raji target cells for 24 h. JeKo-1 cells were rapidly eliminated and did not elicit a CARhi response. [file 12967_2015_519_MOESM7_ESM.pdf]

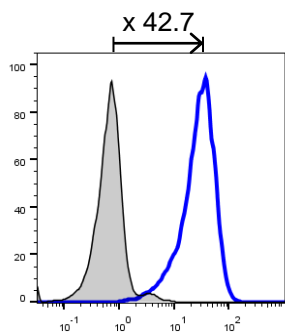

CD19<sup>+</sup> H9

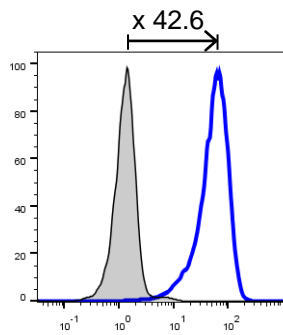

CD19<sup>+</sup> K562

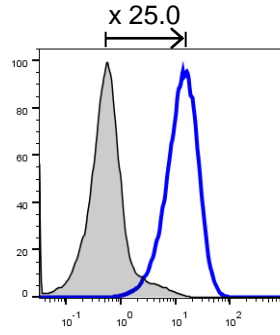

Raji

▣ Anti-CD19 Staining  
 Isotype Control

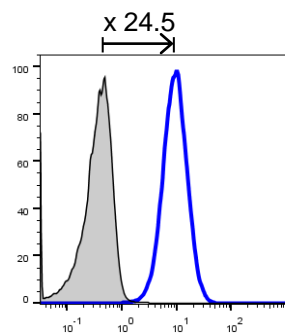

JeKo-1

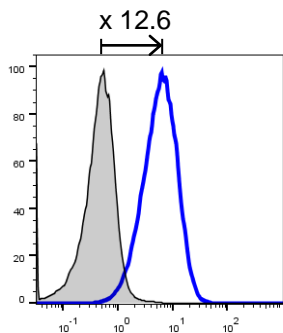

TM-LCL

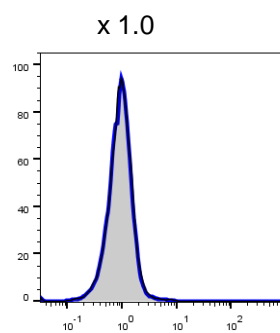

Parental K562

Supplement: Additional file 8: Figure S8. — CD19 expression levels of various target cell lines. CD19+ H9, CD19+ K562, Raji, JeKo-1, TM-LCL, and parental K562 cell lines were stained with an anti-CD19 antibody or an isotype control. Numbers above each plot indicate the fold difference in median fluorescence intensity between CD19 and isotype control staining. [file 12967_2015_519_MOESM8_ESM.pdf]

**A**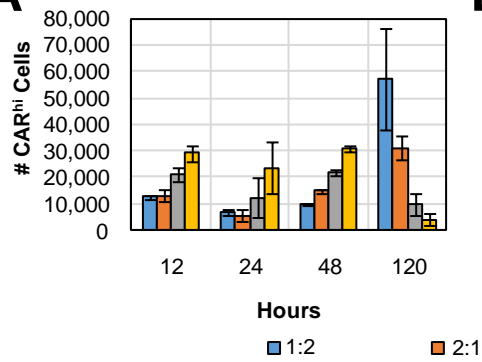**B**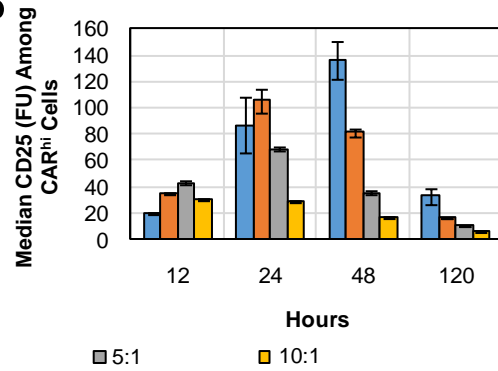

Supplement: Additional file 9: Figure S9. — Stimulating CAR-T cells with varying amounts of irradiated CD19+ K562s impacts the dynamics of the CARhi response. (A) Number of CARhi and (B) median CD25 expression among CARhi cells in a co-incubation of CD8+ CAR-T cells with irradiated CD19+ K562 cells at various E:T ratios. Average values of triplicates are shown with error bars indicating ± 1 s.d. [file 12967_2015_519_MOESM9_ESM.pdf]

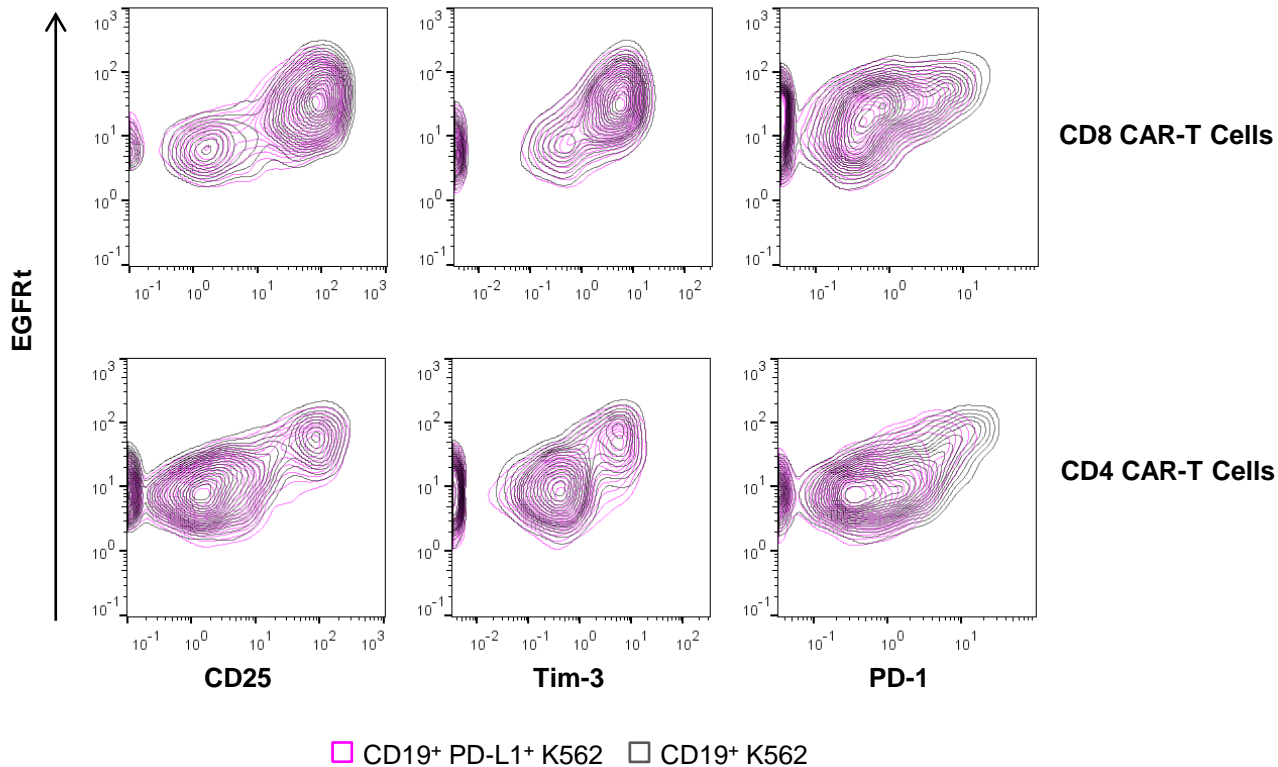

Supplement: Additional file 11: Figure S11. — CARhi cells upregulate CD25, Tim-3, and PD-1 regardless of PD-L1 expression on target cells. CD8+ CAR-T cells were co-incubated with CD19+ or CD19+PD-L1+ target cells without exogenous cytokines and surface-stained with EGFRt, CD25, Tim-3, and PD-1 antibodies. [file 12967_2015_519_MOESM11_ESM.pdf]

**A**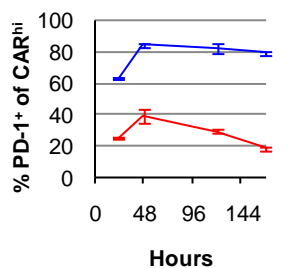**B**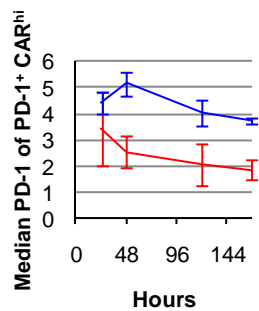

— CD8 CAR

— CD4 CAR

Supplement: Additional file 12: Figure S12. — CD4+ CARhi T cells express higher levels of PD-1 than CD8+ CARhi T cells. CD4+ and CD8+ CAR-T cells were co-incubated with CD19+ target cells without exogenous cytokines and monitored by surface antibody staining for (A) % PD-1+ among CARhi cells and (B) median PD-1 fluorescence among PD-1+ cells. Average values of triplicates are shown with error bars indicating ± 1 s.d. [file 12967_2015_519_MOESM12_ESM.pdf]

**A**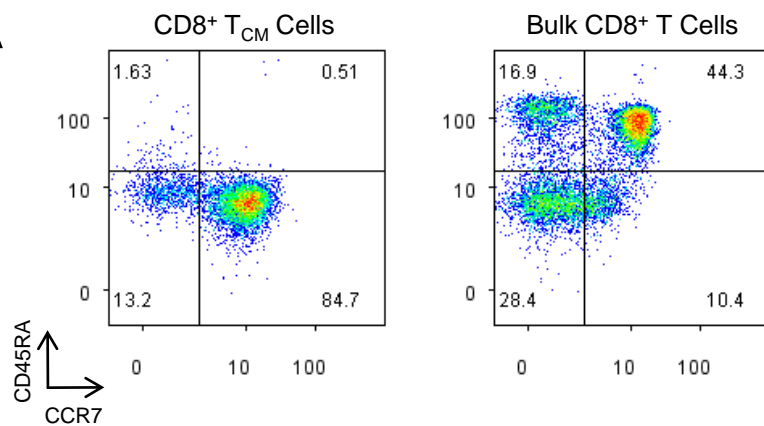**B**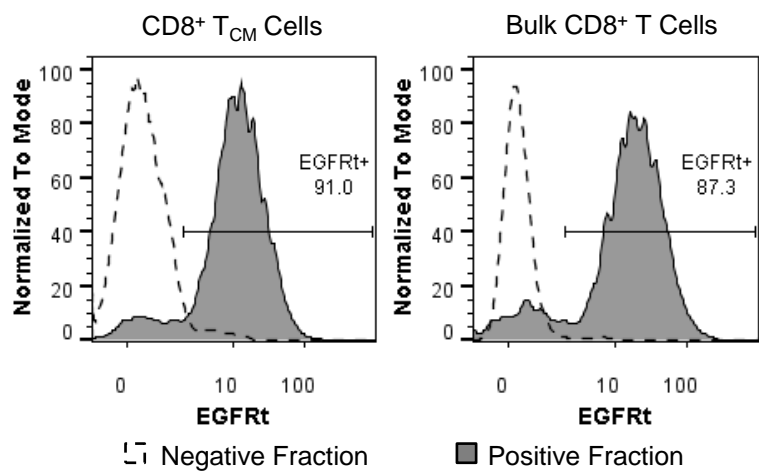

Supplement: Additional file 13: Figure S13. — Isolation of TCM-derived CAR+ cells. (A) The TCM subset (CD45RA−, CCR7+) was enriched from bulk CD8+ T cells. (B) Both TCM and bulk CD8+ cells were transduced with CAR and sorted for the EGFRt+ fraction. Both positive (solid gray) and negative (dotted open) fractions were collected by magnetic bead sorting. Numbers shown indicate the frequency of EGFRt+ cells within the positive fraction. [file 12967_2015_519_MOESM13_ESM.pdf]
