# Supplementary material for: Identification and selective expansion of functionally superior T cells expressing chimeric antigen receptors
Source: J Transl Med. 2015 May 20;13:161. doi: 10.1186/s12967-015-0519-8 (PMC4457995; doi:10.1186/s12967-015-0519-8)
Supplement: Additional file 10: Figure S10. — PD-L1 can be overexpressed on otherwise PD-L1− K562 target cells. (A) Various K562 target cell lines were stained with PD-L1 antibody. (B) K562 lines were also cross-stained for both PD-L1 and CD19 to verify antigen expression. [file 12967_2015_519_MOESM10_ESM.pdf]

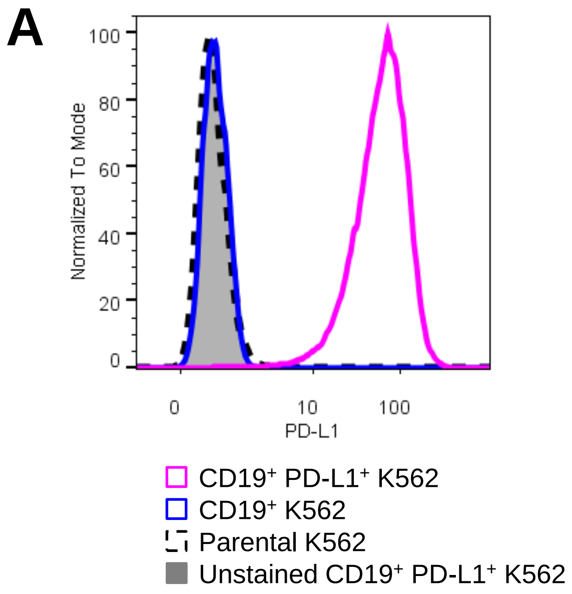
A
Normalized To Mode
100
80
60
40
20
0
0
10
100
PD-L1
CD19⁺ PD-L1⁺ K562
CD19⁺ K562
Parental K562
Unstained CD19⁺ PD-L1⁺ K562
B
PD-L1
100
10
0
0
10
100
CD19
CD19⁺ PD-L1⁺ K562
CD19⁺ K562
Parental K562
