# Supplementary material for: Identification and selective expansion of functionally superior T cells expressing chimeric antigen receptors
Source: J Transl Med. 2015 May 20;13:161. doi: 10.1186/s12967-015-0519-8 (PMC4457995; doi:10.1186/s12967-015-0519-8)
Supplement: Additional file 14: Figure S14. — Phenotypic shifts in the TCM- and bulk-CD8–derived cells. (A) CD45RA and CCR7 surface expression was monitored for TCM-derived cells prior to sorting for EGFRt+ cells, 4 days after sorting (immediately before co-incubation with target cells), and after 36 h of co-incubation with CD19+ target cells. (B) EGFRt and CCR7 surface expression after 36 h of co-incubation with CD19+ target cells. (C) and (D) show the corresponding data for bulk-CD8–derived cells. [file 12967_2015_519_MOESM14_ESM.pdf]

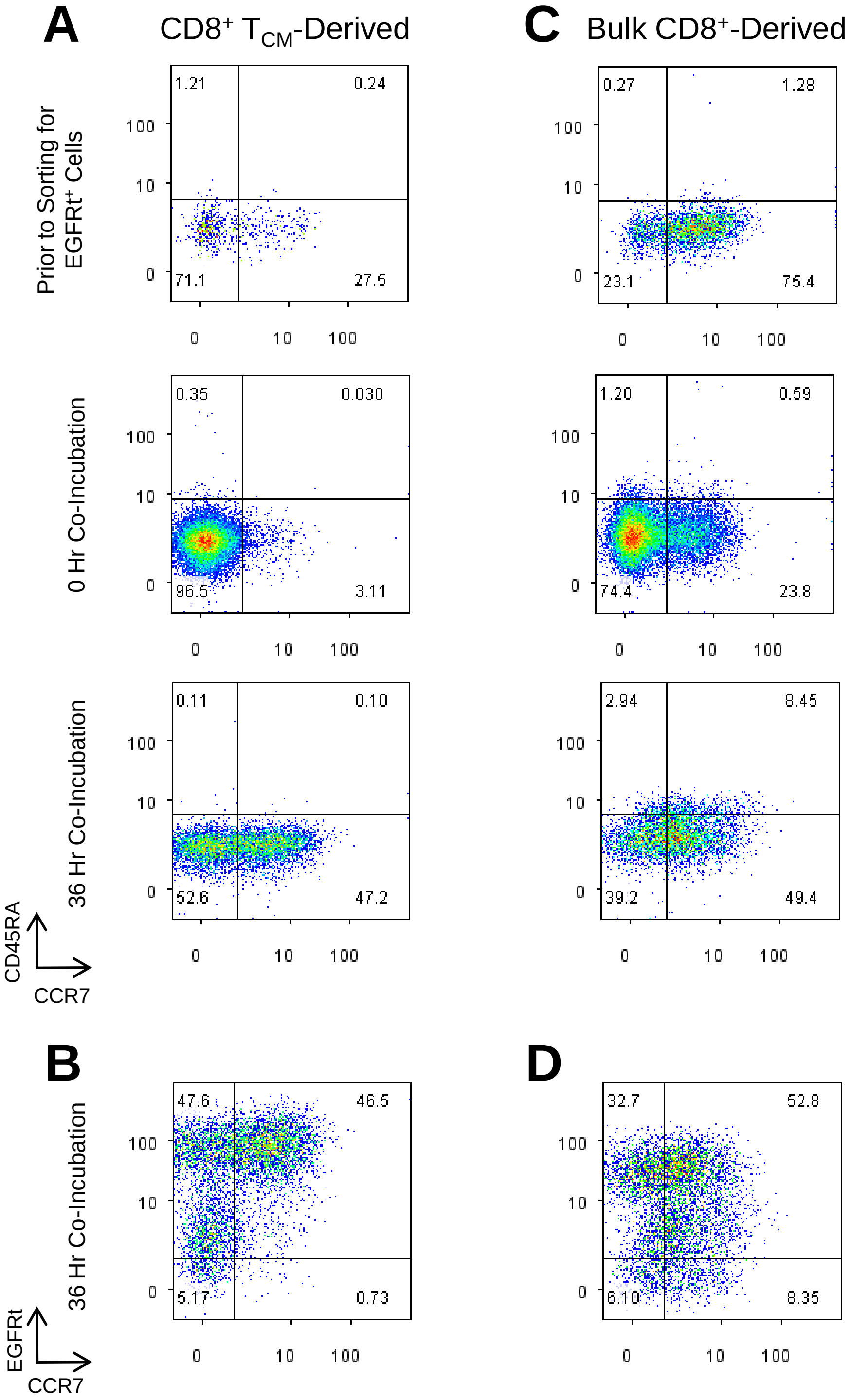

A
CD8+ TCM-Derived
C
Bulk CD8+-Derived
Prior to Sorting for EGFRt+ Cells
0 Hr Co-Incubation
36 Hr Co-Incubation
CD45RA
CCR7
B
D
EGFRt
1.21
0.24
71.1
27.5
0.27
1.28
23.1
75.4
0.35
0.030
96.5
3.11
1.20
0.59
74.4
23.8
0.11
0.10
52.6
47.2
2.94
8.45
39.2
49.4
47.6
46.5
5.17
0.73
32.7
52.8
6.10
8.35
